# Harnessing Epigenetic Modifiers Reveals MAPK-Mediated Regulation Mechanisms in Hadal Fungi of *Alternaria alternata* Under High Hydrostatic Pressure

**DOI:** 10.3390/jof11090650

**Published:** 2025-09-02

**Authors:** Qingqing Peng, Qifei Wei, Xi Yu

**Affiliations:** Shanghai Engineering Research Center of Hadal Science and Technology, College of Oceanography and Ecological Science, Shanghai Ocean University, Shanghai 201306, China

**Keywords:** epigenetic modification, *Alternaria alternata*, high hydrostatic pressure, transcriptomic analysis, regulation mechanism

## Abstract

High hydrostatic pressure (HHP) significantly modulates microbial metabolism, while chemical epigenetic modifiers are known to reactivate silent biosynthetic gene clusters and induce novel natural products. However, the mechanisms by which these epigenetic modifiers regulate fungal responses under differential pressure conditions, and how such regulation affects natural product biosynthesis, remain completely unexplored. Here, we investigated the hadal fungus *Alternaria alternata* CIEL23 isolated from 7332 m sediments in the Mariana Trench under epigenetic modifier treatment with contrasting pressures (0.1 MPa vs. 40 MPa). Our results revealed that epigenetic perturbations and high pressure significantly altered fungal phenotypes, gene expression, and secondary metabolite composition. Transcriptome-level analysis of epigenetic regulatory mechanisms under epigenetic modifiers in both pressure conditions (0.1 MPa and 40 MPa) demonstrated that the addition of epigenetic modifiers regulated MAPK pathway-related gene expression in response to the environment stimuli. Under dual stress conditions, the IG, CWI, and HOG branches of the MAPK pathway showed significantly altered activity patterns. These changes were associated with differential the regulation of genes related to hyphal growth, cell wall remodeling, cell cycle progression, and osmolyte synthesis, suggesting the coordinated modulation of multiple cellular processes. These findings provide the mechanistic link between epigenetic modification induced HHP-response changes and regulation in hadal fungi. Our study not only advances understanding of hadal fungal response to dual stressors but also unlocks new possibilities for harnessing their stress-driven metabolic versatility for biotechnological applications.

## 1. Introduction

The hadal region is the most enigmatic ecosystem on our planet, representing an unparalleled extreme deep-sea environment on Earth. It is characterized by perpetual darkness (aphotic conditions), low temperatures (~3 °C), extreme hydrostatic pressure (≤110 MPa) [1], nutrient scarcity, and topographical isolation/complexity [2,3,4]. The distinctive conditions of the hadal environment may harbor fungi capable of producing specialized natural product structures crucial for enhancing the specificity and efficacy of drugs, reducing toxic side effects, and preventing drug resistance. As one of the extreme characteristics of the habitat, high hydrostatic pressure (HHP) holds significant importance for the study of these organisms. Studies indicate that cultivation under varied pressure regimes triggers fungi to produce secondary metabolites with divergent antimicrobial activities [5]. To date, the regulatory mechanisms governing biosynthetic pathways in hadal fungi under HHP remain poorly understood. This knowledge gap significantly impedes both the exploitation of these unique microbial resources and the elucidation of fundamental biological processes in extreme environments.

Fungi inhabiting distinct environments exhibit unique structural adaptations and specialized natural product profiles [6]. Statistical analyses indicate that approximately 80% of metabolites isolated from marine fungi exhibit diverse bioactivities, including antimicrobial, antitumor, antiviral, enzyme inhibitory, anti-inflammatory, and antioxidant properties [7]. Consequently, marine fungi are recognized as promising sustainable sources for drug discovery [8] and constitute a key reservoir of medicinally valuable secondary metabolites [9]. However, recent years have witnessed the repeated isolation of known compounds from fungal sources, significantly reducing the discovery rate of novel-structure compounds. This trend poses significant challenges to conventional natural product research in fungi.

The application of epigenetic modifiers is a well-established strategy to enhance the chemical diversity of secondary metabolites produced by fungi under laboratory settings [10,11,12]. Recent studies demonstrated that exposure to these agents (e.g., DNA methyltransferase and histone deacetylase inhibitors) can change the structure of chromatin and also influence gene expression [13,14,15]. In fungi, epigenetic regulators facilitate the expression of previously non-expressed or low-expressed genes and activate clusters of biosynthetic genes, leading to the generation of novel natural products that have not been observed previously [16]. Moreover, this phenomenon persists even among fungi inhabiting hadal ecosystems [16]. Interestingly, in addition to triggering novel active compounds, epigenetic regulation is influenced by environmental factors and plays a crucial role in the biological response to environmental stress [17]. Environmental stimuli typically regulate fungal growth, development, and stress responses through modulation of signaling pathways such as the MAPK cascade [18,19]. However, it remains unclear whether epigenetic inhibitors under extreme conditions might induce dual stress effects on filamentous fungi through signal transduction pathways, consequently leading to phenotypic adaptations. This represents a significant gap in the current understanding of fungal stress response mechanisms.

Rational exploitation of hadal fungal resources requires robust molecular mechanistic foundations. In this study, we investigate the epigenetic regulatory responses of a hadal fungus (*Alternaria alternata* CIEL 23) in the presence of chemical epigenetic modifiers under high hydrostatic pressure (40 MPa). By integrating data on phenotypic alterations, we revealed striking differences in gene expression and secondary metabolite production. Furthermore, transcriptomic profiling under HHP conditions uncovered key regulatory mechanisms governing these epigenetic pressure-dependent responses on fungal gene expression. Our findings unlock new possibilities for harnessing their stress-driven metabolic versatility for biotechnological applications and elucidate the molecular mechanisms and biological significance of epigenetic regulation in gene expression control.

## 2. Materials and Methods

### 2.1. Fungual Isolation and Identification

Hadal sediment samples were collected at a depth of 7332 m (60–70 cm below the sediment) from the Mariana Trench (142.2148° E 11.3403° N) during the expedition of the Discovery-One research vessel (TS 21) in November 2019 [5]. The samples and media were prepared following the previous method [20]. The single-spore separation and hyphal-tip purification assays were conducted to obtain a pure culture of the hadal fungi. Fungal isolates were all cultured on potato dextrose agar (PDA, 20% peeled and sliced potato, 1.0% glucose, and 1.5% agar, with deep-sea seawater, natural pH) at 28 °C and stored at 4 °C.

The total fungal genomic DNA was extracted using the TIAN combi DNA Lyse & Det PCR Kit (Tiangen Biotech (Beijing) Co., Ltd., Beijing, China). The fungal internal transcription interval (ITS) sequence was amplified using the universal fungal primers ITS1\4 (5′-TCCGTAGGTGAACCTGCGG-3′\5′-TCCTCCGCTTATTGATATGC-3′) and then the obtained sequence was sequenced by GENEWIZ. The sequencing results were blasted in the NCBI (National Center for Biotechnology Information) database to determine the taxonomy of the isolates.

### 2.2. Quantification of Conidia

To unify the inoculation amount, a constant number of conidial suspensions was prepared before culture. The conidial suspension was quantified as previously described by Li et al. [21]. The obtained hadal fungi were revived on PDA medium for 3 days at 28 °C to obtain fresh mycelium. After that, the fresh mycelia were inoculated on new PDA medium. The inoculated Petri dishes were incubated in a constant temperature incubator at 28 °C for 4 days. Conidia were harvested from the plates with 20 mL of distilled water, and the suspension was filtered through eight layers of gauze to remove the mycelia. The filtrate was appropriately diluted, and then the conidial concentration was determined using a hemocytometer. The prepared conidial suspension was stored at 4 °C.

### 2.3. Culture of Chemical Epigenetic Modifier

The chemical epigenetic modifier 5-Azacytidine (5-AzaC) was selected as a potential modifier, with ddH_2_O as a solvent. The agents were filtered by a 0.22 μm filter membrane and stored at 4 °C. The 1 mL spore suspension (above 2 × 10^4^ spores/mL) described above was inoculated into a 10 mL syringe and treated with the two pressures (0.1 MPa and 40 MPa). Half of the syringes were suspended in a pressure vessel filled with pure water and pressurized to 40 MPa at room temperature. The other half of the syringes were incubated under 0.1 MPa at room temperature as the control. Three replicates were maintained for each treatment. 10 μL conidial suspension was inoculated on the above medium and cultured at 28 °C for 7 days under atmospheric pressure. Colony phenotypes of fungi were observed and recorded every day. Each group was repeated at least three times.

### 2.4. RNA Extraction and Reverse Transcription

Hadal fungi were cultured in potato dextrose broth (PDB, 20% peeled and sliced potato, and 1.0% glucose, with deep-sea seawater, natural pH) medium with different concentrations of 5-AzaC for 10 d at 28 °C, and fresh mycelia were harvested. The harvested mycelia were ground into powder in liquid nitrogen and then used for total RNA isolation. The total RNAs of selected fungi were extracted by the RNAiso Plus reagent (TaKaRa, Dalian, China) according to the manufacturer’s instructions. Degradation and contamination of the total RNAs of hadal fungus were detected by 1% agarose gel electrophoresis. The quantity and purity of total RNA were preliminarily assessed spectrophotometrically based on A260/A280 and A260/A230 ratios measured by the Nano-Drop 2000 UV-vis spectrophotometer (Thermo Scientific, Wilmington, DE, USA). Then, the primeScript RT reagent kit with gDNA Eraser (Perfect Real Time) (TaKaRa, Dalian, China) was used to synthesize cDNA with 1 µg RNA according to the recommended instructions. Total cDNA was stored at −80 °C.

### 2.5. Quantitative Reverse Transcriptase PCR (qRT-PCR)

The PKS gene degenerates primers used in this study were the same as those in previous studies [16], AltqpksF1/AltqpksR1 (5′-GAAAGCGTCACCCTGAAGTA/5′-AAAGGAGGCAGTGGAGCA). The glyceraldehyde-3-phosphate dehydrogenase (GAPDH) gene was employed as the internal reference gene. The qRT-PCR was performed using SYBR Premix Ex Taq II (Tli RNaseH Plus) (TaKaRa, Dalian, China) on the Applied Biosystems 7500 Real-Time PCR System (PerkinElmer Applied Biosystems, Foster City, CA, USA). The PCR mixture (20 µL) contained 10 µL of 2 × SYBR Premix Ex Taq II (Tli RNaseH Plus), 1 µL of cDNA, 0.8 µL of forward and reverse primers, 7 µL of ddH_2_O, and 0.4 µL of 50 × ROX reference dye (TaKaRa, Dalian, China). The PCR reaction was as follows: 30 s at 95 °C, 40 cycles of 5 s at 95 °C, 30 s at 55 °C, and 30 s at 72 °C. Each target gene was amplified using three replicates. Cyclic quantitative (Cq) values were determined based on three biological replicates, each with three technical replicates. The relative quantification of a target molecule relative to the reference gene was performed according to the mean normalized expression (MNE) method [22]. The relative expression was calculated using the 2^−∆∆Ct^ method [23]. All data were analyzed using IBM SPSS Statistics 22 (SPSS 22.0). All the results were expressed as the mean ± standard error (SE). The differences between the variables were evaluated by one-way analysis of variance (ANOVA), followed by the least significant difference (LSD) multiple comparison tests. Compared with blank, results were considered to be significant at the level of *p* (NS *p* > 0.05, * *p* < 0.05, ** *p* < 0.01, *** *p* < 0.001).

### 2.6. Extraction of Secondary Metabolites

The extraction of fungal secondary metabolites was performed using the method described in a previous study with slight modifications [24]. After being cultured on PDB at 28 °C for 10 days on a rotating shaker (180 rpm/min), the mycelia and liquids were separated by vacuum filtration using eight layers of sterilized gauze. The secondary metabolites from the mycelia and liquids were extracted by ultrasonication with an equal volume of ethyl acetate at room temperature. The extraction was repeated three times. All organic components were collected, mixed, and evaporated to dryness using a 45 °C vacuum rotary evaporator. All crude extracts of the fungal cultures were weighed and stored at −20 °C.

### 2.7. UPLC-MS/MS Analysis of Secondary Metabolites

The extracts of hadal fungi were characterized by ultra-performance liquid chromatography/tandem mass spectrometry (UPLC-MS/MS). The crude extracts were dissolved in 1 mL of methanol to the same concentration (100 mg/mL) with methanol and filtered though a 0.22 μm filter membrane. UPLC-MS/MS spectrometric analyses were performed using a Vanquish UPLC high-resolution mass spectrometer (Thermo Fisher) equipped with an electrospray ionization (ESI) source operating in the positive ion and negative mode. A Waters ACQUITY UPLC BEH C 18 column (1.7 μm × 2.1 mm × 100 mm), at a flow rate of 0.4 mL/min, was used for preparative HPLC collection. The column temperature was maintained at 60 °C, and the injection temperature was held at 10 °C. The obtained mass spectrum data were blasted in the COCONUT database, the NP Atlas database, and the StreptomeDB database.

### 2.8. Antimicrobial Activity Assay

The Kirby–Bauer method was used to test the antibacterial properties of the crude extracts against pathogens. There were six strains of pathogenic bacteria provided by Shanghai Rainbowfish Company, including *Staphylococcus aureus* ATCC25923, *Enterococcus faecalis* FA2-2, *Escherichia coli* MG1655, *Chromobacterium violaceum* ATCC12472 CV026, *Salmonella choleraesuis*, and *Mycobacterium smegmatis*, and three strains of aquatic pathogens, including *Edwardsiel latarda*, *Klebsiella pneumoniae*, and *Aeromonas hydrophila*, as the indicated bacteria for this assay. The test sample was quantified to a final concentration of 100 mg/mL with methanol. The indicated pathogens were inoculated in the sterilized Luria–Bertani (LB, 5.0 g yeast extract, 10.0 g tryptophan, 10.0 g sodium chloride, pH adjusted to 7.0) broth medium at 37 °C on a rotary shaker (180 rpm/min) for 16 h. The culture of indicator pathogens (OD about 0.5) was evenly spread with 200 μL of pathogen solution on the LB agar medium. Circular sterile filter papers (6 mm) were placed on the plates. 2 μL of the test sample was dropped onto filter paper, and methanol was used as the negative control. After incubating at 37 °C for 16 h, the diameters of the inhibition zones were measured and compared with the control to determine the antimicrobial activity. The antibacterial rate was calculated to evaluate the antibacterial effect. All the experiments were repeated three times. All data were analyzed using SPSS Statistics 22.

### 2.9. Transcriptome Analysis

To perform RNA sequencing and transcriptomics analysis, hadal fungi were incubated at 180 rpm/min and 28 °C in a PDB medium containing different concentrations of 5-AzaC for two days. Then, the mycelia were transferred to different pressure conditions (0.1 MPa and 40 MPa) for three days, after which the biomass was harvested. The mycelia were thoroughly ground using liquid nitrogen and preserved in RNAiso Plus (Takara, Kyoto, Japan) at −80 °C. The RNA extraction, transcriptomics sequencing, and bioinformatics analysis were accomplished by Beijing NOwo Zhiyuan (BNOZ, Beijing, China). According to the standard guidelines, the validated RNA underwent purification, fragmentation, reverse transcription, end repairing, amplification, and circularization to obtain the library. Quality control was also applied. The final product was loaded onto an Illumina NovaSeq X Plus platform for RNA sequencing (RNA-Seq). Low-quality reads were filtered through an Agilent 2100 bioanalyzer (accurate detection of RNA integrity). Clean reads for subsequent analysis were obtained after raw data filtering, sequencing error rate checking, and GC content distribution checking. The obtained clean reads were spliced using Trinity [25].

To obtain comprehensive gene function information, we annotated the gene function in seven databases, including NCBI non-redundant protein sequences (Nr), NCBI nucleotide sequences (Nt), Protein family (Pfam) [26], euKaryotic Ortholog Groups (KOG), A manually annotated and reviewed protein sequence database (Swiss-prot), Kyoto Encyclopedia of Genes and Genomes (KEGG), and Gene Ontology (GO), for the obtained splicing transcripts. Gene expression levels were calculated as FPKM (expected number of Fragments Per Kilobase of transcript sequence per million base pairs sequenced) using RSEM [27]. Analysis of differentially expressed genes (DEGs) was performed using DESeq2 [28]. |log2 Fold-Change| ≥ 1.0 and padj ≤ 0.05 were set as the criteria for screening DEGs between different groups. By enriching and annotating differential genes in the GO database [29] and the KEGG database [30], biological functions or pathways significantly related to differential genes under different conditions could be found, thus revealing and understanding the basic molecular mechanisms of biological processes.

### 2.10. Transcriptome Validation

To verify the results obtained from RNA-Seq, qRT-PCR analysis was performed as described previously [31]. Total RNA was obtained from BNOZ, and first-strand cDNA was synthesized by the PrimeScript RT Reagent Kit (Takara, Japan) following the manufacturer’s instructions. The relative expression of genes was calculated using the 2^−∆∆Ct^ method. The RNA-Seq fold changes were plotted against the qRT-PCR fold changes to calculate correlation coefficients (R2) [32]. β-tubulin (5′-GGCAAGACCATCCGTTTC/5′-CAGCAGAGGGAGCAGAAAT) was used as a reference gene in this work.

### 2.11. Statistical Analysis

All experiments and groups were performed at least in triplicate. For each sample, three biological replicates were taken. Statistical differences (*p* ≤ 0.05) among the mean quantity of gene expression were determined by ANOVA and Dunnett’s multiple comparisons test in IBM SPSS Statistics 22. The significant difference for all comparisons was set at *p* < 0.05 (NS *p* > 0.05, * *p* < 0.01, ** *p* < 0.01, and *** *p* < 0.001). Data were generally presented as means ± S.D. (standard deviation) with the biological replicates as indicated in each figure legend.

## 3. Results

### 3.1. Epigenetic Responses of Hadal Fungi Under Different HHP Conditions

**Figure 1:**
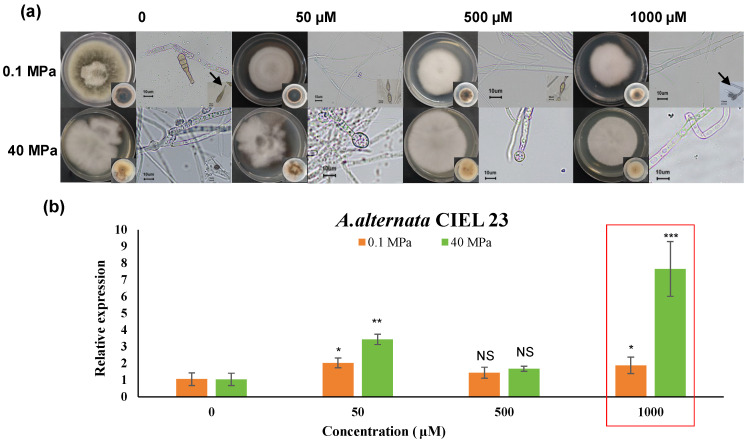
Phenotypes and the PKS gene expression of *A.alternata* CIEL 23. (**a**) the colony phenotypes of *A. alternata* CIEL 23, which was cultured with four concentrations of 5-AzaC (0, 50 μM, 500 μM, and 1000 μM) on PDA at 28 °C under the different culture pressures. The mycelia and spore phenotypes were taken under a ×40 microscope with a scale of 10 μm. The arrow points to the spore elongated beaks. (**b**) the PKS gene expression of *A.alternata* CIEL 23 cultured at different concentrations of epigenetic modifiers under different culture pressures. The *X*-axis was the different concentrations of 5-AzaC, and the *Y*-axis was the relative expression. Compared with black controls, results were considered to be significant at the level of *p* (NS *p* > 0.05, * *p* < 0.05, ** *p* < 0.01, *** *p* < 0.001).

In a previous study, a fungal strain was isolated from hadal sediment collected at 7332 m depth in the Mariana Trench. Based on morphological characteristics and ITS sequence analysis (using primers ITS1/ITS4), the isolate was identified as *Alternaria alternata* and designated as CIEL 23. Colony morphology exhibited regular margins with olive-green pigmentation at the periphery, transitioning to gray at the center, accompanied by a downy, gray texture. Microscopic examination revealed that it developed unbranched conidial chains bearing prominent, elongated beaks at the distal ends, consistent with the typical conidiogenesis phenotype observed in this fungal genus (Figure 1a).

To elucidate the epigenetic regulation of the hadal fungus *A. alternata* CIEL 23 under hydrostatic pressure, cultures were incubated at 28 °C for 10 days under two pressure conditions (0.1 MPa and 40 MPa) with four concentrations (0, 50, 500, and 1000 μM) of the DNA methyltransferase inhibitor 5-AzaC (Table S1). Under both pressures, increasing 5-AzaC concentrations consistently reduced colony expansion and diminished pigmentation on reverse side (Figure 1a).

Since polyketide synthase (PKS) gene expression directly correlates with polyketide biosynthesis capacity [33], we designed specific PKS gene primers targeting the sequence (530–832 bp) with the highest similarity (99.46%) to the PKS gene. Our qPCR analysis revealed that the impact of different concentrations of the 5-AzaC on the expression levels in hadal fungi varied under different culture pressure conditions. Notably, maximal PKS transcript levels were observed at 1000 μM 5-AzaC, showing significant upregulation of 1.78-fold (0.1 MPa) and 7.36-fold (40 MPa) compared to untreated controls (no addition of 5-AzaC), respectively (Figure 1b). This pronounced differential response suggests that hydrostatic pressure substantially influences epigenetic regulation of polyketide biosynthesis in deep-sea fungi.

### 3.2. Impact of Epigenetic Modifiers and High Hydrostatic Pressure on Secondary Metabolite Profiles

**Figure 2:**
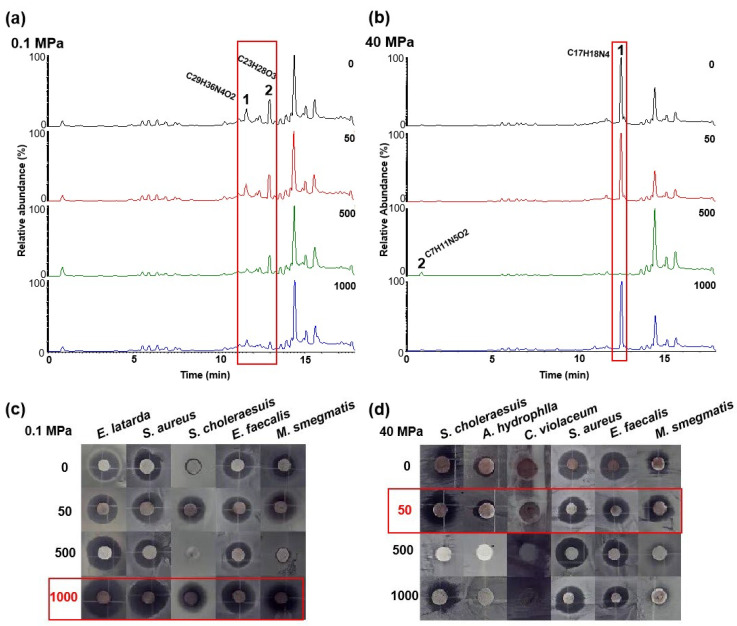
Secondary metabolites of *A. alternata* CIEL 23 at different concentrations of 5-AzaC under different conditions. (**a**) the UPLC-MS/MS spectra of secondary metabolites of *A. alternata* CIEL 23 under atmospheric pressure condition. (**b**) the UPLC-MS/MS spectra of the secondary metabolites of *A. alternata* CIEL 23 under HHP condition. The *X*-axis was the retention time (min), and the *Y*-axis was the Relative response (%). The total ion chromatogram (TIC) of the products produced by the strain in media containing different concentrations of 5-AzaC (the number in the upper right corner of the picture indicates the concentration of 5-AzaC) was indicated by different colored lines (black-0, red-50 μM, green-500 μM, and blue-1000 μM). The different signal peaks were marked in the red box. (**c**) the antibacterial activities of secondary metabolite of *A. alternata* CIEL 23 under atmospheric pressure condition. (**d**) the antibacterial activities of secondary metabolite of *A. alternata* CIEL 23 under HHP condition. The number on the left of the picture indicates the concentration of 5-AzaC. The additive concentration group inducing maximal response was boxed in red.

To evaluate the effect of chemical epigenetic modifiers on the natural products of hadal fungi under different pressure conditions, the crude extracts were profiled by UPLC-MS/MS. We found that 5-AzaC treatment significantly altered the secondary metabolite profile of the hadal fungal strain under different pressure conditions. Under atmospheric pressure condition, the total ion chromatogram (TIC) of the crude extracts from the strain cultured in medium with 5-AzaC at a concentration of 1000 μM showed that the signal intensity of peaks 0.1 MPa-1 (11.6 min) and 0.1 Mpa-2 (13.0 min) were significantly weakened compared to the untreated controls (Figure 2a and Figure S1a). However, the TIC analysis revealed the complete disappearance of peak 40 Mpa-1 (12.5 min) with 500 μM of 5-AzaC, which was detectable in untreated controls, along with the emergence of a new metabolite (40 Mpa-2; 1.0 min). (Figure 2b and Figure S1b). Through blasting in the database, the compounds were identified putatively as 0.1 Mpa-1 (C_29_H_36_N_4_O_2_), 0.1 Mpa-2 (C_23_H_28_O_3_), 40 Mpa-1 (C_17_H_18_N_4_), and 40 Mpa-2 (C_7_H_11_N_5_O_2_).

In addition, six strains of human pathogens and three strains of aquatic pathogens were chosen to conduct antibacterial activities of the CIEL 23 cultured with different concentrations of 5-AzaC by the Kirby–Bauer method. Our results demonstrated that HHP conditions and the addition of epigenetic modifiers (5-AzaC) influenced the antibacterial activity of secondary metabolites of *A. alternata* CIEL 23. Under atmospheric pressure, the crude extract at 500 μM 5-AzaC exhibited antibacterial activity against only four pathogens (*M. smegmatis*, *E. faecalis*, *S. aureus*, and *E. ictaluri*), with inhibition rates of 14.3%, 53.8%, 53.8%, and 60.9%, respectively. Notably, he crude extract supplemented with 1000 μM 5-AzaC exhibited maximal antibacterial activity, inhibiting five pathogenic strains (*E. ictaluri*, *S. aureus*, *S. choleraesuis*, *E. faecalis*, and *M. smegmatis*) with respective inhibition rates of 58.1%, 63.3%, 52.6%, 68.4%, and 67.3% (Figure 2c and Figure S2a). However, under HHP conditions, it seems that low-concentration modifiers were more capable of promoting the antibacterial effect of crude extracts. The 50 μM treatment exhibited maximal efficacy, inhibiting six pathogenic strains (*M. smegmatis*, *E. ictaluri*, *S. aureus*, *C. violaceum*, *A. hydrophila*, and *S. choleraesuis*) with respective inhibition rates of 58.1%, 57.1%, 64.0%, 18.2%, 28.0%, and 60.0%. In contrast, the 1000 μM treatment only inhibited four pathogens (*M. smegmatis*, *E. faecalis*, *S. aureus*, and *S. choleraesuis*) at lower efficiencies (28.0%, 58.1%, 60.9%, and 51.4%, respectively) (Figure 2d and Figure S2b).

### 3.3. Transcriptome Sequence Analysis and De Novo Assembly

To further investigate the epigenetic regulatory mechanisms of hadal fungi under different HHP conditions at the molecular level, transcriptomic profiles of *A. alternata* CIEL 23 across treatment groups were analyzed via RNA sequencing. This study focused on the effect of 1000 μM 5-AzaC under two pressures (0.1 MPa and 40 MPa) by comparing the fungal response to epigenetic modifiers. Transcriptome sequencing of the four treatment groups (three replicates per treatment) was performed on the Illumina NovaSeq X Plus platform of Beijing NOwo Zhiyuan (BNOZ, China), including de novo assembly and sequence analysis.

A total of 12 cDNA libraries were constructed: A (atmospheric blank group), B (atmospheric experimental group with 5-AzaC added), C (HHP treatment group), and D (HHP experimental group with 5-AzaC added). Raw sequencing data were processed by removing adapter sequences, filtering undetermined editing information of reads, and discarding low-quality reads (Qphred ≤ reads with 20 bases accounting for more than 50% of the entire read length). After stringent quality control and data screening, a total of 75.52 Gb of clean data were obtained, and the percentage of Q30 bases of each sample was no less than 94.0%. The error rate of each sample was 0.01%. With Trinity [25] for assembly, a sum of 27,786 transcripts and 10,389 unigenes were acquired. The N50 of transcript and unigene reached 25,872 and 18,785, respectively (Table S2).

### 3.4. Functional Annotation Analysis

To annotate expressed unigenes and transcripts, we aligned the assembled sequences with seven known databases (Nr, Nt, KO, Swiss-Prot, Pfam, GO, KOG). Annotation results revealed that 9251 genes (89.04% of the total) were annotated in at least one database (Figure S3).

According to the GO database, the annotated genes were categorized into three primary domains: biological process (BP), cellular component (CC), and molecular function (MF). The GO annotation results indicated that those genes were enriched for cellular process (3719), metabolic process (3408), and biological regulation (1552) in the BP. It seemed that the main areas of enrichment for the genes in the MF were binding (3560), catalytic activity (3126), and transporter activity (980). Furthermore, in the CC, the main areas of enrichment for the genes were cellular anatomical entity (2910), protein-containing complex (1464), and virion component (252) (Figure S4).

The KOG database annotation identified only 107 gene clusters associated with secondary metabolite biosynthesis, whereas significantly more genes were linked to posttranslational modification, protein turnover, and chaperones (496) (Figure S5). After KO annotation, genes were mapped to the KEGG metabolic pathways (Figure S6), which primarily represented genetic information processing (1573), metabolism (394), signal transduction, and cellular processes (768) related to protein families.

### 3.5. Analysis of Expression Differences and Enrichment

The co-expression Venn diagram depicted uniquely expressed genes per sample group, with overlapping regions indicating co-expressed genes across multiple groups. Screening used |FC| ≥ 2 and Padj < 0.05 acted as the screening criteria. To be specific, FC denotes the ratio of expression levels between two groups. As could be seen from Figure S7, there were 2911 genes related to the basic life activities of *A. alternata* CIEL 23 (co-expressed genes of four groups). Furthermore, the different treatment groups possessed unique differentially expressed genes (DEGs).

**Figure 3:**
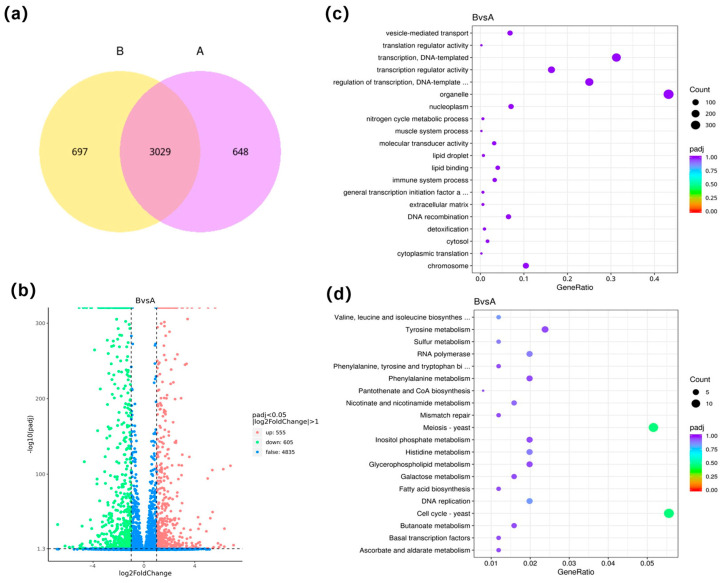
Graph of differentially expressed genes analyzed in group B\A. (**a**) the Venn diagram of gene co-expression. The graphs showed the number of genes uniquely expressed in each group, and the overlapping areas showed the number of genes co-expressed in two groups. (**b**) the volcano map of differential genes. The *X*-axis was the log2Fold Change value, and the *Y*-axis was the −log10 padj or −log10 *p*-value. The blue dashed line indicated the threshold line for the differential gene screening criteria. (**c**) the scatter plot of GO pathway enrichment. (**d**) the scatter plot of KEGG pathway enrichment. In (**c**,**d**), the *X*-axis was the Gene Ratio, and the *Y*-axis was the name of the pathway. The size of the padj was indicated by the color of the dots. The smaller the padj was, the closer the color was to red. The number of differential genes contained under each pathway was indicated by the size of the dots.

**Figure 4:**
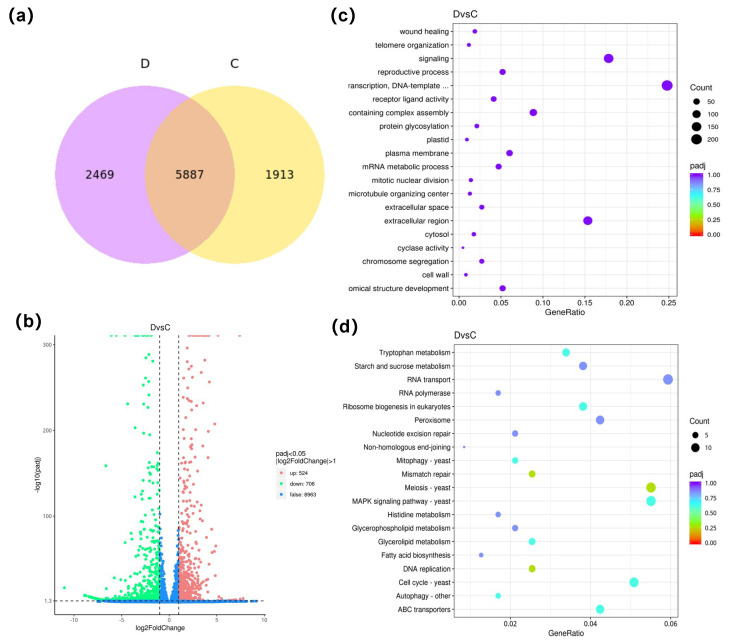
Graph of differentially expressed genes analyzed in group D\C. (**a**) the Venn diagram of gene co-expression. The graphs showed the number of genes uniquely expressed in each group, and the overlapping areas showed the number of genes co-expressed in two groups. (**b**) the volcano map of differential genes. The *X*-axis was the log2Fold Change value, and the *Y*-axis was the −log10 padj or −log10 *p*-value. The blue dashed line indicated the threshold line for the differential gene screening criteria. (**c**) the scatter plot of GO pathway enrichment. (**d**) the scatter plot of KEGG pathway enrichment. In (**c**,**d**), the *X*-axis was the Gene Ratio, and the *Y*-axis was the name of the pathway. The size of the padj was indicated by the color of the dots. The smaller the padj was, the closer the color was to red. The number of differential genes contained under each pathway was indicated by the size of the dots.

When the concentration of 5-AzaC was considered as a single variable, a total of 3029 genes were co-expressed in group B\A under atmospheric pressure conditions. Specifically, 648 genes were exclusively expressed in group A, and 697 genes were uniquely expressed in group B (Figure 3a). A total of 1160 DEGs were screened, of which 555 (47.84%) were upregulated and 605 (52.16%) were downregulated (Figure 3b). The GO enrichment analysis manifested that the primary functions of the DEGs were associated with organelle (368), transcription (266), and transcriptional regulation (213) (Figure 3c). The KEGG enrichment analysis suggested that the majority of those DEGs were predominantly in the cell cycle (14) and meiosis (13) (Figure 3d). A total of 5887 genes were co-expressed in group D\C under HHP conditions. Specifically, 2469 genes were solely expressed in group D, and 1913 genes were exclusively expressed in group C (Figure 4a). A sum of 1230 DEGs was screened. Of which 524 (42.60%) were upregulated and 706 (57.40%) were downregulated (Figure 4b). The GO annotation results indicated that the primary functions of the DEGs were associated with transcriptional regulation (210), signaling (151), and the extracellular region (130) (Figure 4c). The KEGG enrichment analysis suggested that the majority of those DEGs were predominantly in RNA transport (14), Meiosis (13), and MAPK signaling pathway (13) (Figure 4d).

To further verify the RNA-Seq data, RT-qPCR was applied to test the relative expression levels of eight key DEGs (Table S3, Figure S8). The qRT-PCR results were consistent with the RNA-Seq data and further demonstrated the reliability of transcriptome sequencing results.

### 3.6. Epigenetic Modifier Regulated the MAPK Signaling Pathway in HHP

Previous studies have established the critical role of the MAPK signaling pathway in regulating fungal morphogenesis and cell cycle responses to environmental cues [18,19]. In this study, we observed significant upregulation of genes involved in the MAPK pathway in fungi with epigenetic modifiers under HHP (D\C), whereas no changes were detected with epigenetic modifiers under atmospheric pressure conditions B\A (Figure S9).

**Figure 5:**
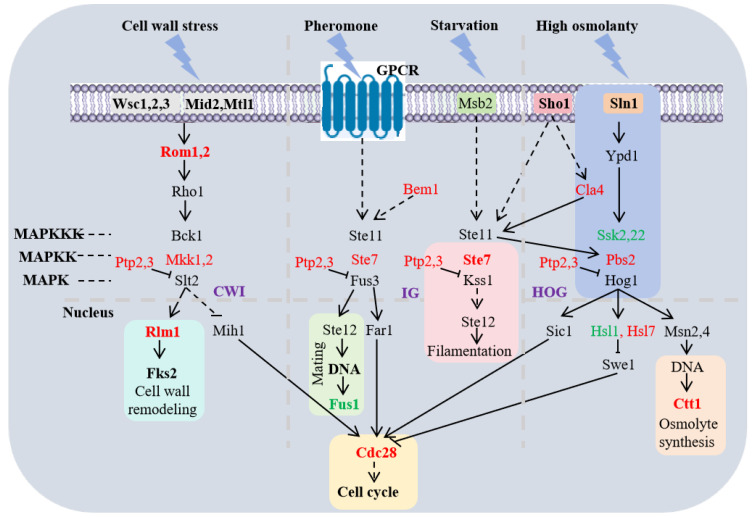
Diagram of the MAPK signaling pathway. In the figure, the upregulation was marked in red, and the downregulation was marked in green. The solid lines represent direct effects, and the dotted lines represent indirect effects. The arrows represent promotion, and the vertical lines represent inhibition.

A total of 18 MAPK-related DEGs were enriched under HHP conditions, comprising three upregulated and fifteen downregulated genes within the MAPK pathway (ko04011). KEGG enrichment analysis revealed that these DEGs were primarily associated with inhibiting cell wall remodeling, filamentation, osmolyte synthesis, cell cycle, and mating. However, the addition of 5-AzaC led to the enrichment of fifteen MAPK-related DEGs, with twelve upregulated and three downregulated genes in the MAPK pathway. KEGG analysis showed that these genes were primarily associated with the cell wall remodeling, cell cycle and osmotic synthesis (Figure 5). These results indicated that the chemical epigenetic modifier (5-AzaC) reduced DNA methylation levels and regulated the expression of most genes in the MAPK signaling pathway under HHP conditions.

### 3.7. IG-MAPK Pathway Responsed to Epigenetic Modification Though Genes Involved in Filamentation, Mating and Cell Cycle

In plant pathogens, the invasive growth (IG) pathway is often indirectly involved in fungal responses to various environmental stresses [34]. Transcriptome results indicated that *Ste7* (MAPKK), a gene associated with the filamentation pathway, was significantly upregulated in the IG pathway following 5-AzaC treatment in both B/A and D/C groups (Figure 5).

**Figure 6:**
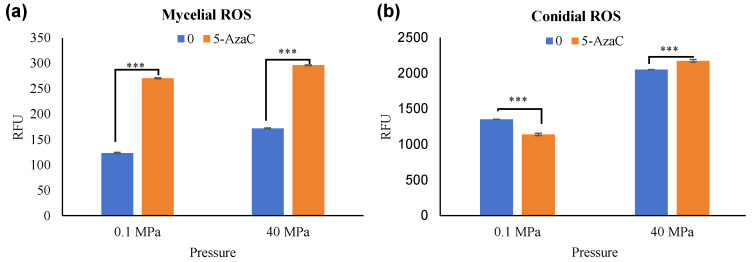
Diagram of reactive oxidative stress in mycelia and conidia. (**a**) the ROS of mycelia. (**b**) the ROS of conadia. The *X*-axis was the pressure of the cultured, and the *Y*-axis was the absorbance value detected by the fluorescent microplate reader. Compared with black controls, results were considered to be significant at the level of *p* (*** *p* < 0.001).

Reactive oxidative stress (ROS) can act as signaling molecules to activate stress response pathways, aiding hyphal adaptation to adverse environments [35]. In this study, the ROS levels of mycelia and spores were quantitatively determined using the Reactive Oxygen Species Assay Kit (Beyotime, Shanghai, China) (Figure 6). Under dual-stress conditions with 5-AzaC, hyphal ROS levels increased twofold, whereas spore ROS remained unchanged. This suggested that hyphae were more sensitive to environmental changes than spores.

Epigenetic variation can be inherited through mitosis or meiosis [36]. The *Cdc28*, which regulates DNA synthesis and mitosis in the cell cycle (ko04111), showed upregulated following 5-AzaC treatment in both B/A and D/C groups. These results demonstrated that chemical epigenetic modifiers could regulate gene expression involved in the IG-MAPK pathway response to external stimuli.

### 3.8. CWI-MAPK Pathway Responsed to Epigenetic Modification Though Genes Involved in Cell Wall Remdeling and Cell Cycle Under HHP Conditions

The cell wall integrity (CWI) MAPK pathway is necessary for the remodeling of the fungal cell wall during growth and development and in response to environmental stimuli, which is highly conserved in the fungal kingdom [37]. Our transcriptome analysis revealed that when cell wall integrity was impaired, sensor proteins (*Mid2*, *Wsc1–3*, and *Mfl1*) transduced extracellular stress signals. In the 5-AzaC-treated HHP group (D/C group), the expression levels of the genes *Rom1*, *2*, and *Rlm1* involved in the pathways related to cell wall remodeling in the fungal cells were all increased (Figure 5). While cell wall damage induced by 5-AzaC triggered upregulation of all remodeling pathway components (*Rom1/2-Rlm1-Fks2*), this response was absent in the atmospheric group B/A. These results demonstrated that chemical epigenetic modifiers regulated CWI-MAPK pathway gene expression under HHP.

**Figure 7:**
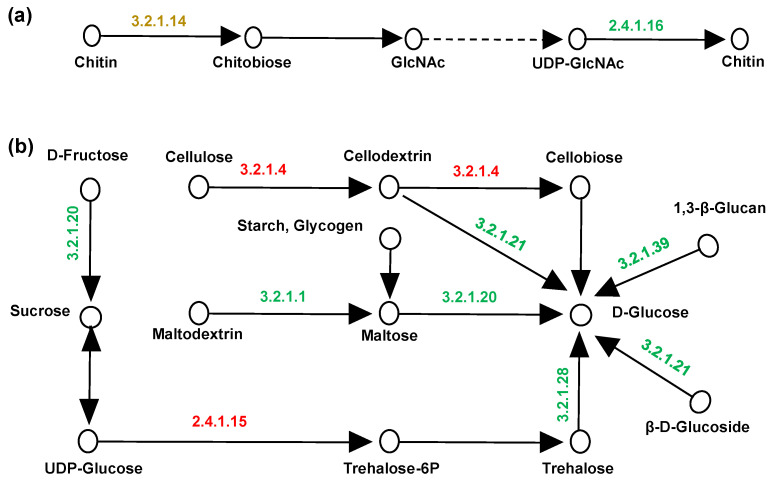
Diagram of the pathway of chitin metabolism and starch and sugar metabolism. (**a**) the chitin metabolism in D\C group. (**b**) the starch and sugar metabolism in D\C group. The upregulation was marked in red, the downregulation was marked in green, and both were marked in yellow. The arrow indicates the direction of synthesis.

Transcriptomic analysis of amino acid, sugar, and nucleotide metabolism (ko00520) and starch and sugar metabolism (ko00500) demonstrated altered expression of cell wall biosynthesis genes in 5-AzaC-treated fungi under HHP conditions. Specifically, chitin biosynthesis was inhibited (Figure 7a), while genes for glucose, maltose, and fructose production were downregulated. Conversely, cellulose degradation and trehalose-6-phosphate (T6P) synthesis genes were upregulated (Figure 7b). These transcriptional changes suggested structural modifications in fungal cell walls following DNA hypomethylation in HHP environments.

### 3.9. HOG-MAPK Pathway Responsed to Epigenetic Modification Though Osmolyte Synthesis and Cell Cycle Under HHP Conditions

The high-osmolarity glycerol (HOG) MAPK pathway, which specifically mediates osmotic stress responses, is regulated by two upstream branches (*Sln1* and *Sho1*) that exhibit distinct functional roles but typically act through negative feedback [38,39,40]. Our transcriptomic analysis revealed that in hadal fungi, adding 5-AzaC downregulated the osmotic stress sensing gene *Sln1* and *Ssk2,22* in both B/A and D/C groups (Figure 5).

The *Sho1* branch shares multiple components with other pathways, such as oxidative and heat stress [41,42]. Although *Sho1* expression did not change significantly in the D\C group, its downstream (*Cla4-Ste11-Pbs2-Hog1*) was activated. This was a phenomenon absent in the B\A group. Moreover, after culturing hadal fungi under HHP conditions, mycelial swelling in medium containing different 5-AzaC concentrations correlated markedly with upregulation expression of *Hsl7*, which interacts with *Hog1* to regulate the cell cycle via morphogenetic checkpoints, and upregulated the osmolyte synthesis gene *Ctt1*. These genetic alterations indicated that chemical epigenetic modifiers could remodel the cell cycle and osmotic response via the HOG pathway to cope with HHP conditions.

## 4. Discussion

Epigenetic modifiers have been demonstrated to significantly influence fungal metabolic processes and secondary metabolite production, thereby stimulating the diversification of natural products [43,44,45,46]. However, current research has predominantly focused on standard laboratory conditions, and their specific regulatory mechanisms under extreme environments remain poorly understood, which limits One Strain Many Compounds (OSMAC) applications in extremophilic fungi [15]. DNA methylation is a key epigenetic modification that influences gene expression, and its reduction can lead to the activation of previously silenced genes [47]. In this study, we investigated the effects of high hydrostatic pressure (40 MPa) combined with the chemical epigenetic modifier (5-AzaC) in hadal fungi *A. alternata* CIEL 23. Our findings revealed that 5-AzaC significantly increased the expression of the PKS gene in the hadal fungi under 40 MPa culture conditions. Transcriptome analysis of samples from four experimental groups showed that hadal fungi regulated other cell functions mainly by influencing changes in the MAPK signaling pathway within cells, thereby helping them adapt to environmental changes. Further studies should focus on elucidating these mechanisms to expand the discovery of novel bioactive compounds from extremotolerant fungi.

DNA methyltransferase (DNMT) inhibition by 5-AzaC induces passive demethylation through consecutive DNA replication cycles [48]. Transcriptome analysis also indicated that under different pressure culture conditions, only in the experimental group with 5-AzaC added were the cell cycle-related genes (*Cdc28*) of hadal fungi significantly accelerated. This result demonstrated that the demethylation induced by chemical epigenetic modifiers in hadal fungi was not associated with environmental stress. Epigenetic regulation does not operate in isolation but is highly integrated into environmental signaling networks [17]. The MAPK signaling pathway is involved in various stress responses, and its activation enhances the adaptability of fungi to oxidative stress, osmotic changes, and other environmental challenges [49,50]. In the dual stress groups, the gene expression levels of genes related to cell wall remodeling, filamentation, and osmolyte synthesis throughout the MAPK signaling pathway were upregulated. This phenomenon has not been observed in any previous studies regarding the effects of HHP on fungi. The decrease in DNA methylation not only increased the potential of fungi to adapt to environmental changes but also served as the foundation for rapid gene expression regulation and swift responses to environmental changes. In this study, we demonstrated that under different pressure environment conditions, the addition of DNA methyltransferase inhibitor (5-AzaC) not only reduced the degree of methylation of fungal DNA but also induced the environment-dependent differential regulation of the MAPK signaling pathway.

The other two interesting points were found in our transcriptomic data. One of them was that the cell wall components of fungi in HHP environments undergo alterations. Firstly, the cell wall exhibited elasticity rather than rigidity and demonstrated rapid structural reorganization that could influence cellular survival following osmotic shock [51]. Additionally, the cell wall serves as a fundamental structural element in fungi, capable of dynamic reshaping in response to variations in temperature, pH, and oxidative or osmotic stress [52,53,54]. Hyphae could more effectively regulate septal numbers to enhance hardness for withstanding expansion pressures at 20 MPa [41]. However, this strategy became ineffective beyond 40 MPa. High-pressure conditions can induce the expression of the ABC transport system in deep-sea bacteria, thereby increasing the content of branched-chain fatty acids in membrane lipid composition and maintaining the fluidity of the cell membrane [55]. Here, in the dual stress groups, the hadal fungus reduced the expression of genes related to chitin, sugar, and cellulose in cell walls, while upregulating the expression of genes related to ABC transport proteins. These putative structural changes might be the result of the effects of epigenetic modifiers on fungi in HHP environments. Another intriguing aspect was the shift in energy metabolism and cell cycle among fungi under HHP conditions. Analysis of sugar metabolism changes D\C revealed that when environmental pressure was altered, the expression of various genes related to carbohydrate consumption within cells was upregulated. However, upon introducing 5-AzaC at 40 MPa, the gene expression in intracellular utilization pathways such as glucose, fructose, and maltose was downregulated, which indicates the epigenetic regulation under HHP conditions. Furthermore, the upregulated gene expression levels of genes related to the cell cycle suggest that the fungal cells may be reallocating resources to maintain cellular integrity under HHP conditions. The cell cycle is tightly regulated to ensure proper growth and division, and its modulation under stress conditions is critical for cellular adaptation [56]. While our study provides new insights, its limitations in elucidating these two aspects highlight the need for further exploration and validation.

The physiological processes of fungi are extremely complex, involving the interaction of multiple signaling pathways and regulatory mechanisms. Although the results of this study confirmed the influence of chemical epigenetic modifiers on the gene expression of hadal fungi under HHP conditions. However, the situation in the actual hadal environment may be more complex. Unfortunately, our study is more about the description of gene expression changes and transcriptomic results, as well as putative mechanisms based on existing research results. In the future, we will be able to find evidence that directly proves the regulation of fungi under extreme environmental conditions. Based on the current limitations of fungal research in hadal environments, this study holds significant theoretical and practical importance and will also provide new ideas for exploring the life processes and evolutionary mechanisms of hadal organisms.

## Data Availability

Data are contained within the article and Supplementary Materials.

## Supplementary Materials

The following supporting information can be downloaded at: https://www.mdpi.com/article/10.3390/jof11090650/s1. Figure S1: UPLC-MS/MS diagram of secondary metabolites of *A. alternata* CIEL 23. Figure S2: Statistical diagram of the secondary metabolite activities of *A. alternata* CIEL 23. Figure S3: Venn diagram of annotation results in 5 databases. Figure S4: Categorical statistical chart of GO annotated. Figure S5: Categorical statistical chart of KOG annotated. Figure S6: Categorical statistical chart of KEGG annotated. Figure S7: Venn diagram of gene co-expression. Figure S8: Statistical diagram of the relative expression of the gene validating RNA-Seq results. Figure S9: Diagram of the MAPK signaling pathway in B\A group. Table S1: Table of the media containing different concentrations of chemical epigenetic modifiers used in this experiment. Table S2: Summary of RNA-seq results. Table S3: Table of qRT-PCR primers to validate RNA-Seq results.

## Abbreviations

The following abbreviations are used in this manuscript:

HHP	high hydrostatic pressure
PDA	potato dextrose agar
NCBI	National Centre for Biotechnology Information
5-AzaC	5-Azacytidine
PDB	potato dextrose broth
qRT-PCR	quantitative reverse transcriptase PCR
GAPDH	glyceraldehyde-3-phosphate dehydrogenase
Cq	cyclic quantitative
MNE	mean normalized expression
SE	standard error
ANOVA	one-way analysis of variance
LSD	least significant difference
UPLC-MS/MS	ultra-performance liquid chromatography/tandem mass spectrometry
ESI	electrospray ionization
LB	Luria–Bertani
BNOZ	Beijing NOwo Zhiyuan
RNA-Seq	RNA sequencing
Nr	NCBI non-redundant protein sequences
Nt	NCBI nucleotide sequences
Pfam	Protein famil
KOG	EuKaryotic Ortholog Groups
Swiss-prot	Protein sequence database
KEGG	Kyoto Encyclopedia of Genes and Genomes
GO	Gene Ontology
DEGs	differentially expressed genes
PKS	polyketide synthase
IG	invasive growth
ROS	reactive oxidative stress
CWI	cell wall integrity
T6P	trehalose-6-phosphate
HOG	high-osmolarity glycerol
OSMAC	One Strain Many Compounds
DNMT	DNA methyltransferase

## References

[1] Etter R.J., Grassle J.F. (1992). Patterns of species diversity in the deep sea as a function of sediment particle size diversity. Nature.

[2] Arístegui J., Gasol J.M., Duarte C.M., Herndld G.J. (2009). Microbial oceanography of the dark ocean’s pelagic realm. Limnol. Oceanogr..

[3] Debbab A., Aly A.H., Lin W.H., Proksch P. (2010). Bioactive compounds from marine bacteria and fungi. Microb. Biotechnol..

[4] Guo C., Yang Z., Chen J., Zhang D. (2018). Ran, L. Wang, C. Lu, B. Cheng, Q. Study on food sources and trophic levels of benthic organisms in Yap Trench based on stable carbon and nitrogen isotopes. Acta Oceanol. Sin..

[5] Peng Q., Li Y., Deng L., Fang J., Yu X. (2021). High hydrostatic pressure shapes the development and production of secondary metabolites of Mariana Trench sediment fungi. Sci. Rep..

[6] Zhao C., Liu H., Zhu W. (2016). New natural products from the marine-derived Aspergillus fungi-A review. Wei Sheng Wu Xue Bao Acta Microbiol. Sin..

[7] Holler U., Konig G.M., Wright A.D. (1999). Three new metabolites from marine-derived fungi of the genera coniothyrium and microsphaeropsis. J. Nat. Prod..

[8] Xue C., Wang G., Jin S., Zheng T. (2004). Advances in Marine Microbial Diversity Research. Adv. Mar. Sci..

[9] Rosario N., Antonio T. (2016). Bioactive compounds produced by strains of Penicillium and Talaromyces of marine origin. Mar. Drugs.

[10] Ibrahim S.R., Abdallah H.M., Mohamed G.A., Deshmukh S.K. (2023). Exploring potential of Aspergillus sclerotio rum: Secondary metabolites and biotechnological rel evance. Mycol. Prog..

[11] Kamat S., Kumar S., Philip S., Kumari M. (2023). Secondary metabolites from marine fungi: Current status and application. Microbial Biomolecules.

[12] Tiwari P., Bae H. (2022). Endophytic fungi: Key insights, emerging prospects, and challenges in natural product drug discovery. Microorganisms.

[13] Li X., Xia Z., Tang J., Wu J., Tong J., Li M., Ju J., Chen H., Wang L. (2017). Identification and Biological Evaluation of Secondary Metabolites from Marine Derived Fungi-*Aspergillus* sp. SCSIOW3, Cultivated in the Presence of Epigenetic Modifying Agents. Molecules.

[14] VanderMolen K.M., Darveaux B.A., Chen W.-L., Swanson S.M., Pearce C.J., Oberlies N.H. (2014). Epigenetic Manipulation of a Filamentous Fungus by the Proteasome-Inhibitor Bortezomib Induces the Production of an Additional Secondary Metabolite. RSC Adv..

[15] Williams R.B., Henrikson J.C., Hoover A.R., Lee A.E., Cichewicz R.H. (2008). Epigenetic remodeling of the fungal secondary metabolome. Org. Biomol. Chem..

[16] Peng Q., Li Y., Fang J., Yu X. (2023). Effects of Epigenetic Modification and High Hydrostatic Pressure on Polyketide Synthase Genes and Secondary Metabolites of Alternaria alternata Derived from the Mariana Trench Sediments. Mar. Drugs.

[17] Rispail N., Soanes D.M., Ant C., Czajkowski R., Grünler A., Huguet R., Perez-Nadales E., Poli A., Sartorel E., Valiante V. (2009). Comparative genomics of MAP kinase and calcium-calcineurin signalling components in plant and human pathogenic fungi. Fungal Genet. Biol..

[18] Khan A., Shah S.T., Basit A., Mohamed H.I., Li Y. (2024). Mitogen-Activated Protein Kinase: A Potent Signaling Protein that Combats Biotic and Abiotic Stress in Plants. J. Plant Growth Regul..

[19] Lin L., Wu J., Jiang M., Wang Y. (2021). Plant mitogen-activated protein kinase cascades in environmental stresses. Int. J. Mol. Sci..

[20] Raghukumar C., Muraleedharan U., Gaud V.R., Mishra R. (2004). Xylanases of marine fungi of potential use for biobleaching of paper pulp. J. Ind. Microbiol. Biotechnol..

[21] Li Y.F., Sun T.T., Guo D.G., Gao J., Zhang J.A., Cai F., Fischer R., Shen Q.R., Yu Z.Z. (2021). Comprehensive analysis of the regulatory network of blue-light-regulated conidiation and hydrophobin production in Trichoderma guizhouense. Environ. Microbiol..

[22] Simon P. (2003). Q-Gene: Processing quantitative real-time RT-PCR data. Bioinformatics.

[23] Gnat S., Łagowski D., Nowakiewicz A. (2020). Major Challenges and Perspectives in the Diagnostics and Treatment of Dermatophyte nfections. J. Appl. Microbiol..

[24] Scott Chialvo C.H., Griffin L.H., Reed L.K., Ciesla L. (2020). Exhaustive extraction of cyclopeptides from Amanita phalloides: Guidelines for working with complex mixtures of secondary metabolites. Nat. Ecol. Evol..

[25] Grabherr M.G., Haas B.J., Yassour M., Levin J.Z., Thompson D.A., Amit I., Adiconis X., Fan L., Raychowdhury R., Zeng Q.D. (2011). Full-length transcriptome assembly from RNA-Seq data without a reference genome. Nat. Biotechnol..

[26] Finn R.D., Tate J., Mistry J., Coggill P.C., Sammut S.J., Hotz H.R., Ceric G., Forslund K., Eddy S.R., Sonnhammer E.L.L. (2008). The Pfam protein families database. Nucleic Acids Res..

[27] Li B., Dewey C.N. (2011). RSEM: Accurate transcript quantification from RNA-Seq data with or without a reference genome. BMC Bioinform..

[28] Love M.I., Huber W., Anders S. (2014). Moderated estimation of fold change and dispersion for RNA-Seq data with DESeq2. Genome Biol..

[29] Young M.D., Wakefield M.J., Smyth G.K., Osh lack A. (2010). Gene ontology analysis for RNA-seq: Accounting for selection bias. Genome Biol..

[30] Kanehisa M., Araki M., Goto S., Hattori M., Hirakawa M., Itoh M., Katayama T., Kawashima S., Okuda S., Tokimatsu T. (2008). KEGG for linking genomes to life and the environment. Nucleic Acids Res..

[31] Peidro-Guzmán H., Pérez-Llano Y., González-Abradelo D., Fernández-López M.G., Dávila-Ramos S., Aranda E., Olicón-Hernández D., García A., Lira-Ruan V., Pliego O. (2020). Transcriptomic analysis of polyaromatic hydrocarbon degradation by the halophilic fungus Aspergillus sydowii at hypersaline conditions. Environ. Microbiol..

[32] Wang H., Zhang Y., Bartlett D.H., Xiao X. (2021). Transcriptomic analysis reveals common adaptation mechanisms under different stresses for moderately piezophilic bacteria. Microb. Ecol..

[33] Hertweck C. (2009). The biosynthetic logic of polyketide diversity. Angew. Chem. Int. Ed..

[34] He P., Wang Y., Wang X., Zhang X., Tian C. (2017). The mitogen-activated protein kinase CgMK1 governs appressorium formation, melanin synthesis, and plant infection of Colletotrichum gloeosporioides. Front. Microbiol..

[35] Mittler R., Vanderauwera S., Suzuki N., Miller G., Tognetti V., Vandepoele K., Gollery M., Shulaev V., Van Breusegem F. (2011). ROS signaling: The new wave?. Trends Plant Sci..

[36] Peng H., Zhang J. (2009). Stress and plant DNA methylation: Potential applications and challenges in breeding. Adv. Nat. Sci..

[37] Dichtl K., Samantaray S., Wagener J. (2016). Cell wall integrity signalling in human pathogenic fungi. Cell. Microbiol..

[38] Hohmann S. (2009). Control of high osmolarity signalling in the yeast Saccharomyces cerevisiae. FEBS Lett..

[39] Stojanovski K., Ferrar T., Benisty H., Uschner F., Delgado J., Jimenez J., Solé C., de Nadal E., Klipp E., Posas F. (2017). Interaction dynamics determine signaling and output pathway responses. Cell Rep..

[40] Nishimura A., Yamamoto K., Oyama M., Kozuka-Hata H., Saito H., Tatebayashi K. (2016). Scaffold protein Ahk1, which associates with Hkr1, Sho1, Ste11, and Pbs2, inhibits cross talk signaling from the Hkr1 osmosensor to the Kss1 mitogen-activated protein kinase. Mol. Cell Biol..

[41] Jamalzadeh S., Pujari A.N., Cullen P.J. (2020). A Rab escort protein regulates the MAPK pathway that controls filamentous growth in yeast. Sci. Rep..

[42] Zhong M., Li Y., Deng L., Fang J., Yu X. (2023). Insight into the adaptation mechanisms of high hydrostatic pressure in physiology and metabolism of hadal fungi from the deepest ocean sediment. mSystems.

[43] Scherlach K., Hertweck C. (2021). Mining and unearthing hidden biosynthetic potential. Nat. Commun..

[44] Begani J., Lakhani J., Harwani D. (2018). Current strategies to induce secondary metabolites from microbial biosynthetic cryptic gene clusters. Ann. Microbiol..

[45] Nielsen K.F., Larsen T.O. (2015). The importance of mass spectrometric dereplication in fungal secondary metabolite analysis. Front. Microbiol..

[46] Toghueo R., Sahal D., Boyom F.F. (2020). Recent advances in inducing endophytic fungal specialized metabolites using small molecule elicitors including epigenetic modififiers. Phytochemistry.

[47] Pocas-Fonseca M.J., Cabral C.G., Manfrao-Netto J.H.C. (2020). Epigenetic manipulation of filamentous fungi for biotechnological applications: A systematic review. Biotechnol. Lett..

[48] Feil R., Fraga M. (2012). Epigenetics and the environment: Emerging patterns and implications. Nat. Rev. Genet..

[49] Zhang M., Zhang S. (2022). Mitogen-activated protein kinase cascades in plant signaling. J. Integr. Plant Biol..

[50] Pitzschke A., Schikora A., Hirt H. (2009). MAPK cascade signalling networks in plant defence. Curr. Opin. Plant Biol..

[51] Gow N.A.R., Latgé J.P., Munro C.A. (2017). The fungal cell wall: Structure, biosynthesis, and function. Microbiol. Spectr..

[52] Pontón J. (2008). The fungal cell wall and the mechanism of action of anidulafungin. Rev. Iberoam. Micol..

[53] Garcia-Rubio R., de Oliveira H.C., Rivera J., Trevijano-Contador N. (2019). The fungal cell wall: Candida, cryptococcus, and Aspergillus species. Front. Microbiol..

[54] Mouriño-Pérez R.R. (2013). Septum development in filamentous ascomycetes. Fungal. Biol. Rev..

[55] Wang F., Xiao X., Ou H.-Y., Gai Y., Wang F. (2009). Role and regulation of fatty acid biosynthesis in the response of Shewanella piezotolerans WP3 to different temperatures and pressures. J. Bacteriol..

[56] Peter J. (2012). Functions of DNA methylation: Islands, start sites, gene bodies and beyond. Nature reviews. Genetics.

