# Supplementary material for: Harnessing Epigenetic Modifiers Reveals MAPK-Mediated Regulation Mechanisms in Hadal Fungi of Alternaria alternata Under High Hydrostatic Pressure
Source: J Fungi (Basel). 2025 Sep 2;11(9):650. doi: 10.3390/jof11090650 (PMC12470266; doi:10.3390/jof11090650)
Supplement: Supplementary file 1 [file jof-11-00650-s001.zip › jof-3791632-supplementary.pdf]

Harnessing epigenetic modifiers reveals MAPK-mediated regulation mechanisms in hadal fungi of *Alternaria alternata* under high hydrostatic pressure

Qingqing Peng, Qifei Wei and Xi Yu *

Shanghai Engineering Research Center of Hadal Science and Technology, College of Oceanography and Ecological Science, Shanghai Ocean University, Shanghai 201306, China;

d220200047@st.shou.edu.cn (Q.P.); m230200676@st.shou.edu.cn.

* Correspondence: xyu@shou.edu.cn.

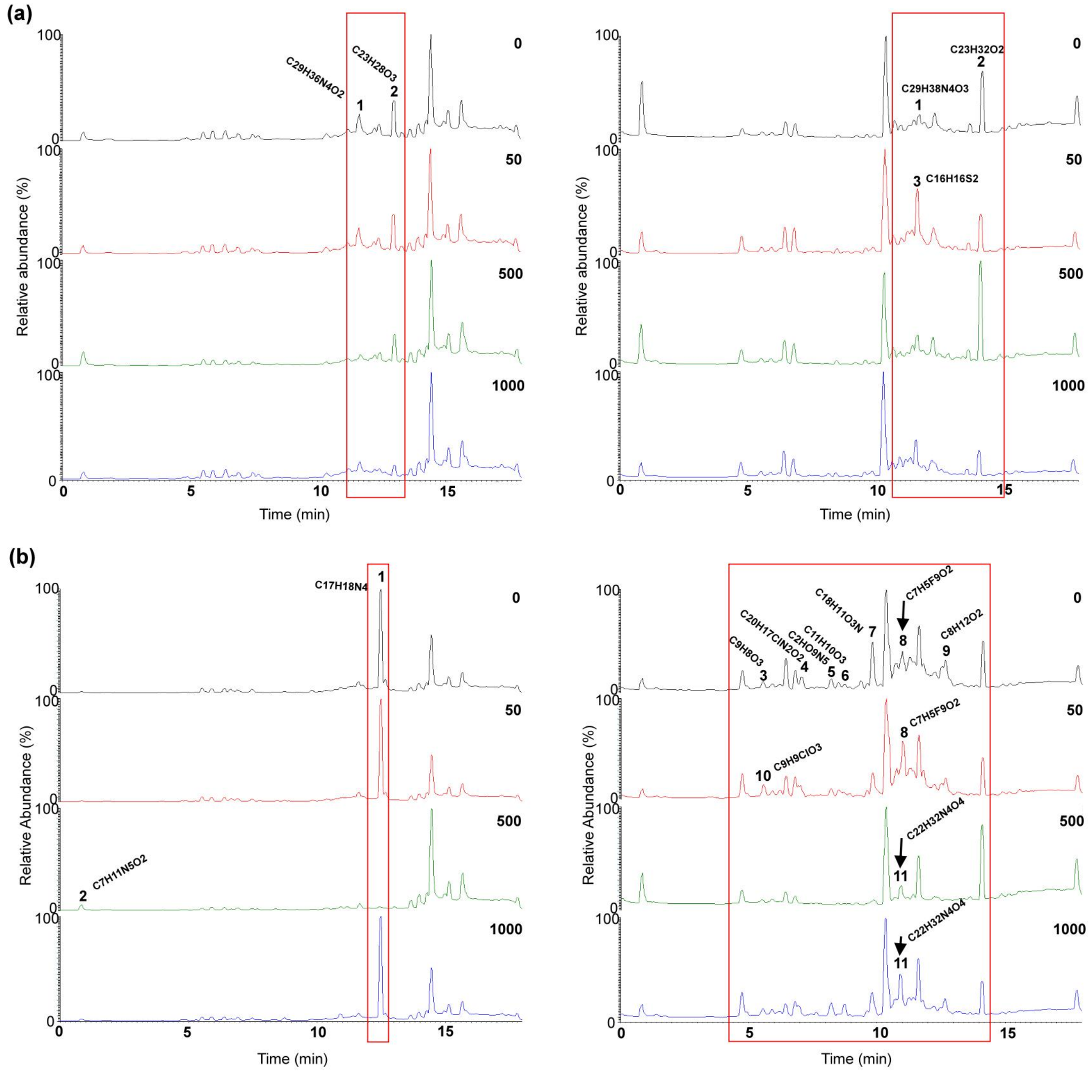

Figure S1 UPLC-MS/MS diagram of secondary metabolites of *A.alternata* CIEL 23. (a) *A.alternata* CIEL 23 cultured under 0.1 MPa with different concentrations of 5-AzaC. (b) *A.alternata* CIEL 23 cultured under 40 MPa with different concentrations of 5-AzaC. The X-axis was the retention time (min), and the Y-axis was the Relative response (%). The total ion chromatogram (TIC) of the products produced by the strain in media containing different concentrations of 5-AzaC (the number in the upper right corner of the picture indicates the concentration of 5-AzaC) was indicated by different colored lines (black-0, red-50 μM, green-500 μM, and blue-1000 μM). The different signal peaks were marked in the red box.

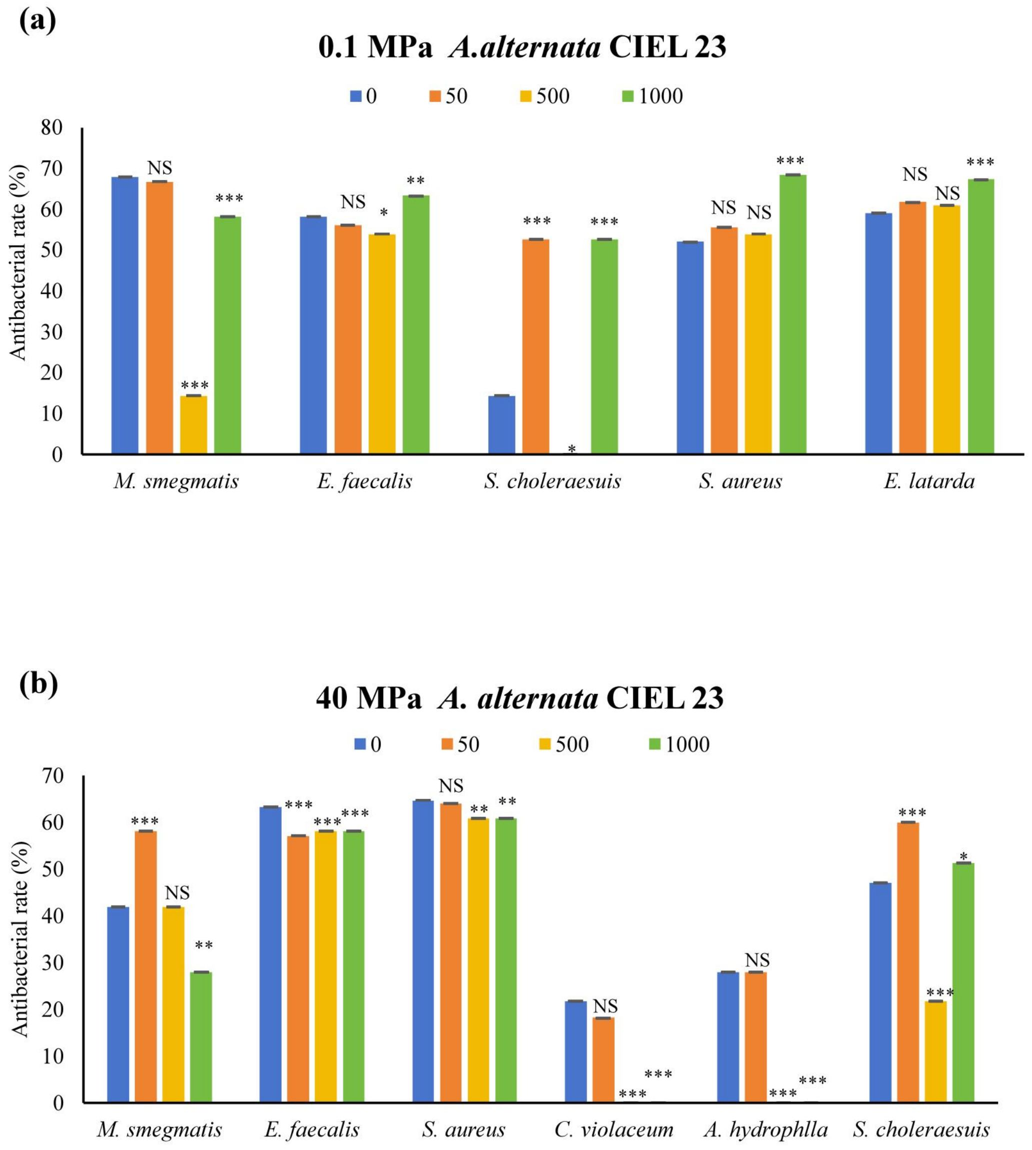

Figure S2 Statistical diagram of the secondary metabolite activities of *A.alternata* CIEL 23. (a) the inhibitory rate calculated according to the diameter of the inhibitory zone of *A.alternata* CIEL 23 cultured under 0.1 MPa. (b) the inhibitory rate calculated according to the diameter of the inhibitory zone of *A.alternata* CIEL 23 cultured under 40 MPa. Error bars indicated standard deviation (SD). Compared with black control, results were considered to be significant at the level of p (NS $p > 0.05$, * $p < 0.05$, ** $p < 0.01$, *** $p < 0.001$).

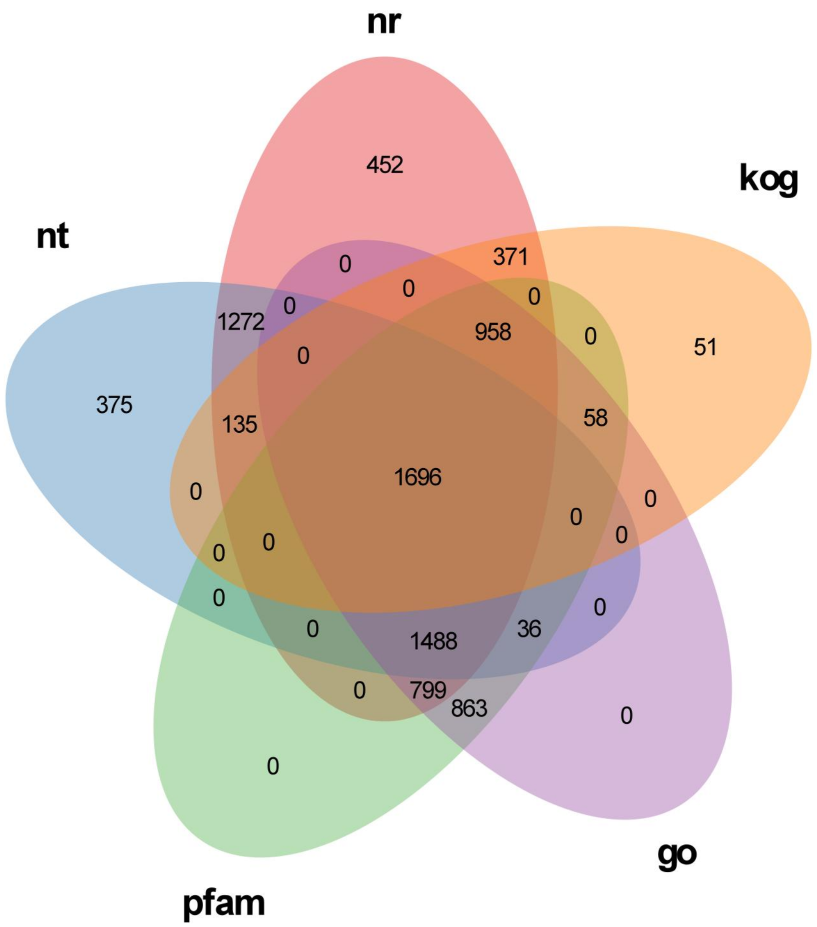

Figure S3 Venn diagram of annotation results in 5 databases. Different colors indicated different databases.

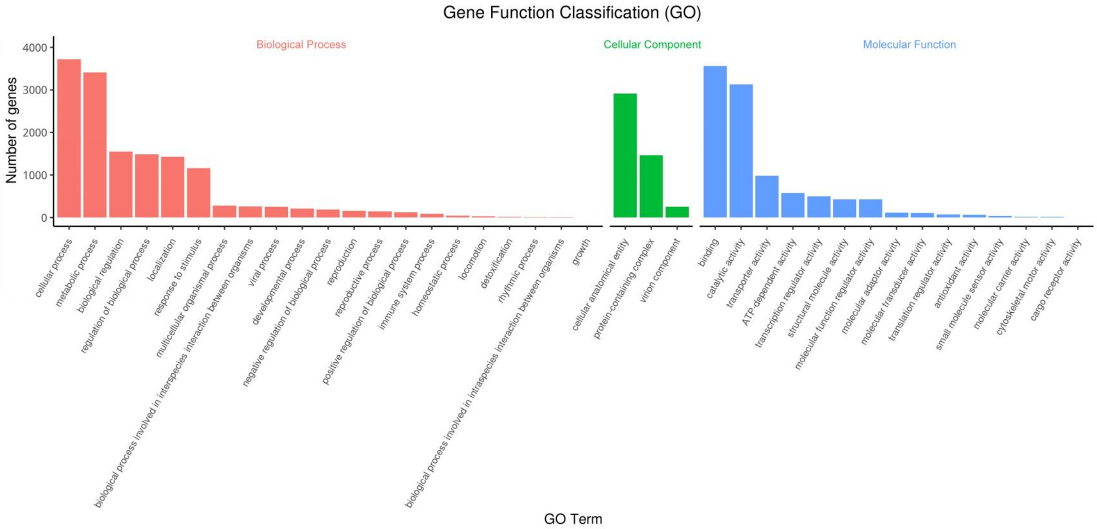

Figure S4 Categorical statistical chart of GO annotated. The X-axis was the GO Term, and the Y-axis was the number of genes annotated to that GO Term. In the figure, the red part presented the biological process, the green part presented the cellular component, and the blue part presented the molecular function.

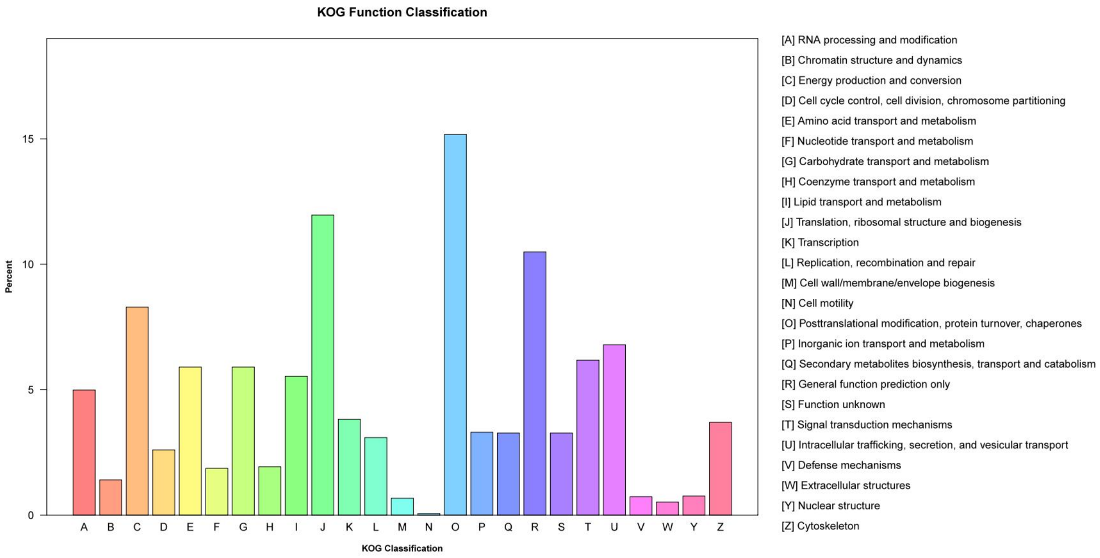

Figure S5 Categorical statistical chart of KOG annotated. The X-axis was the name of the KOG classification, and the Y-axis was the ratio of the number of genes annotated to this group to the total number of genes annotated in it.

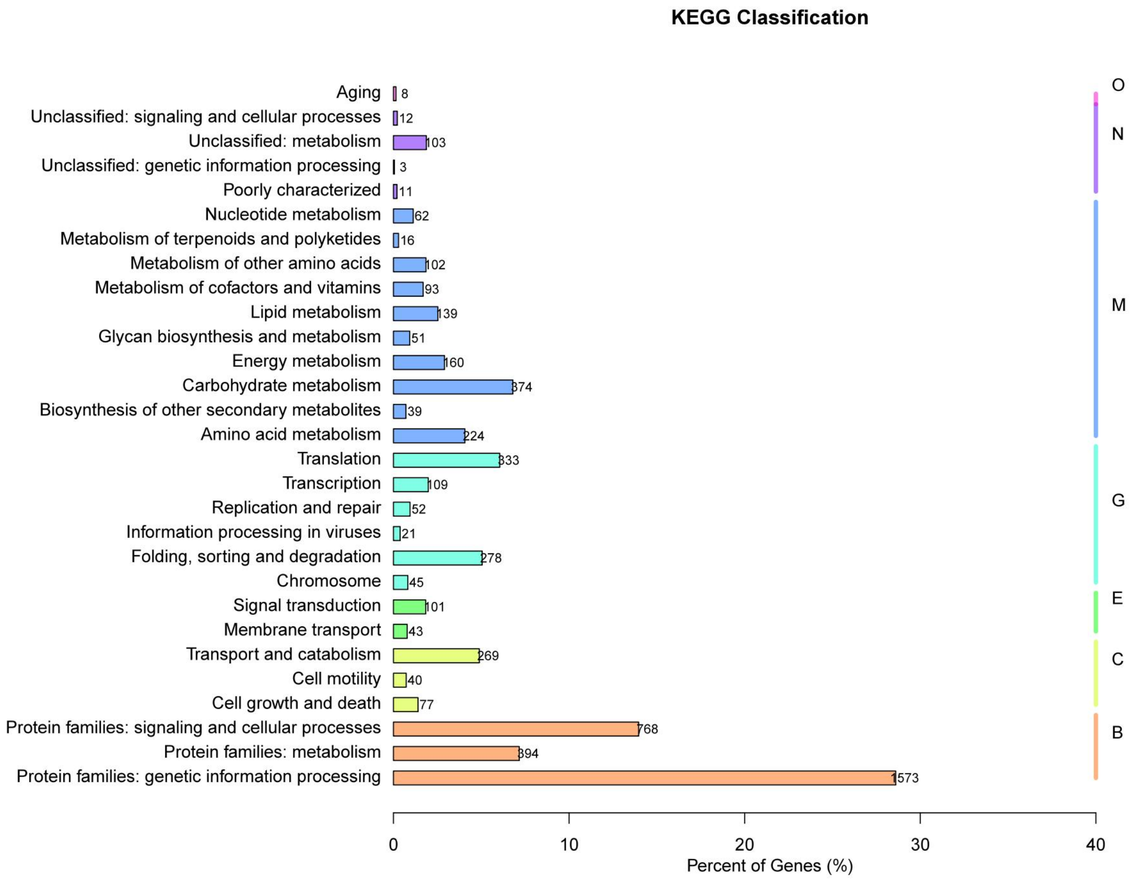

Figure S6 Categorical statistical chart of KEGG annotated. The X-axis was the number of genes annotated under the pathway and their number as a proportion of the total number of genes on which they were annotated, and the Y-axis was the names of KEGG metabolic pathways.

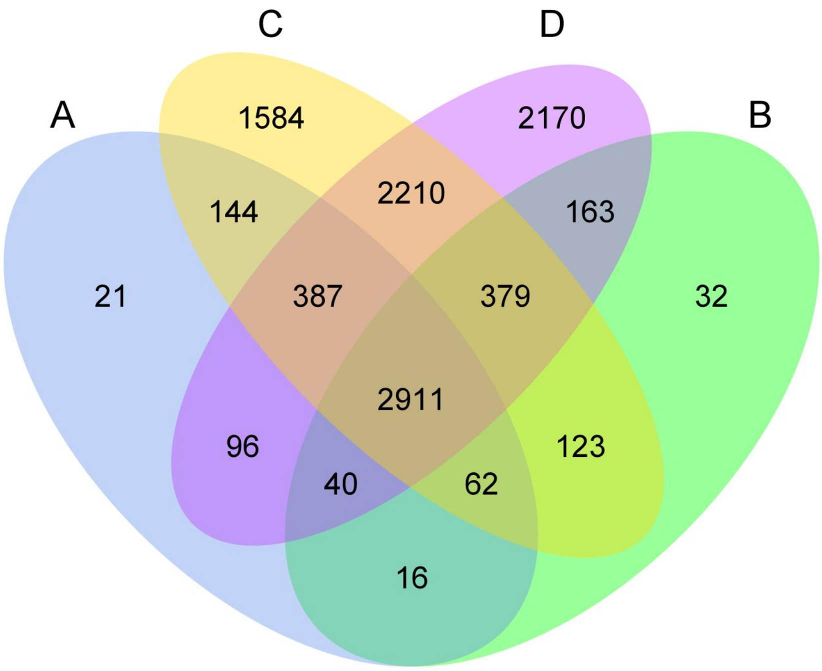

Figure S7 Venn diagram of gene co-expression. In this figure, A represents the atmospheric blank group. B represents the atmospheric experimental group. C represents the HHP blank group. D represents the HHP experimental group. The overlapping regions showed the number of genes co-expressed in two or more groups of samples.

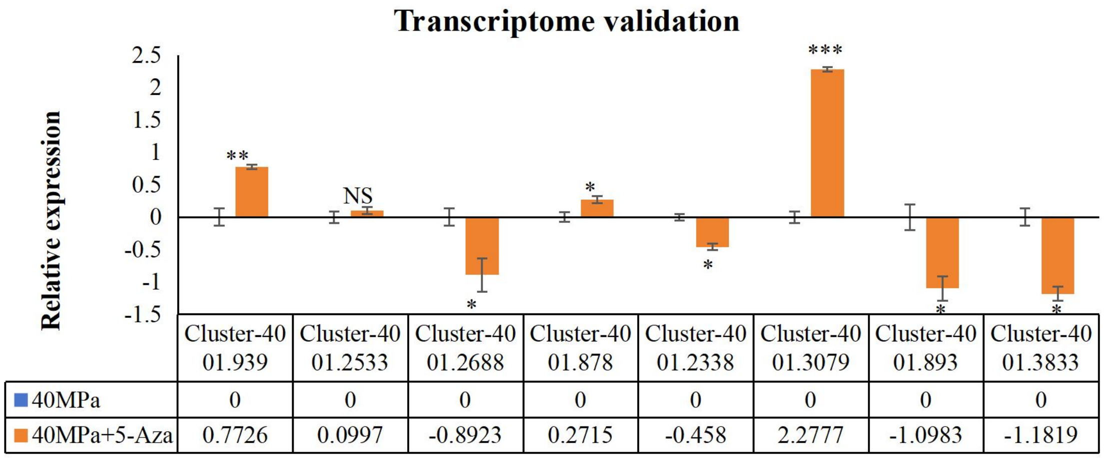

	Cluster-40 01.939	Cluster-40 01.2533	Cluster-40 01.2688	Cluster-40 01.878	Cluster-40 01.2338	Cluster-40 01.3079	Cluster-40 01.893	Cluster-40 01.3833
40MPa	0	0	0	0	0	0	0	0
40MPa+5-Aza	0.7726	0.0997	-0.8923	0.2715	-0.458	2.2777	-1.0983	-1.1819

Figure S8 Statistical diagram of the relative expression of the gene validating RNA-Seq results. The X-axis was the gene ID, and the Y-axis was the relative expression. The $2^{-\triangle\triangle Ct}$ method was adopted to calculate the expression difference multiples between the treatment group and the blank group with β-tublin as the internal reference gene. The relative expression of genes and the log2FC value in the RNA-Seq results were compared. Compared with 40 MPa, results were considered to be significant at the level of p (NS $p > 0.05$, * $p < 0.05$, ** $p < 0.01$, *** $p < 0.001$).

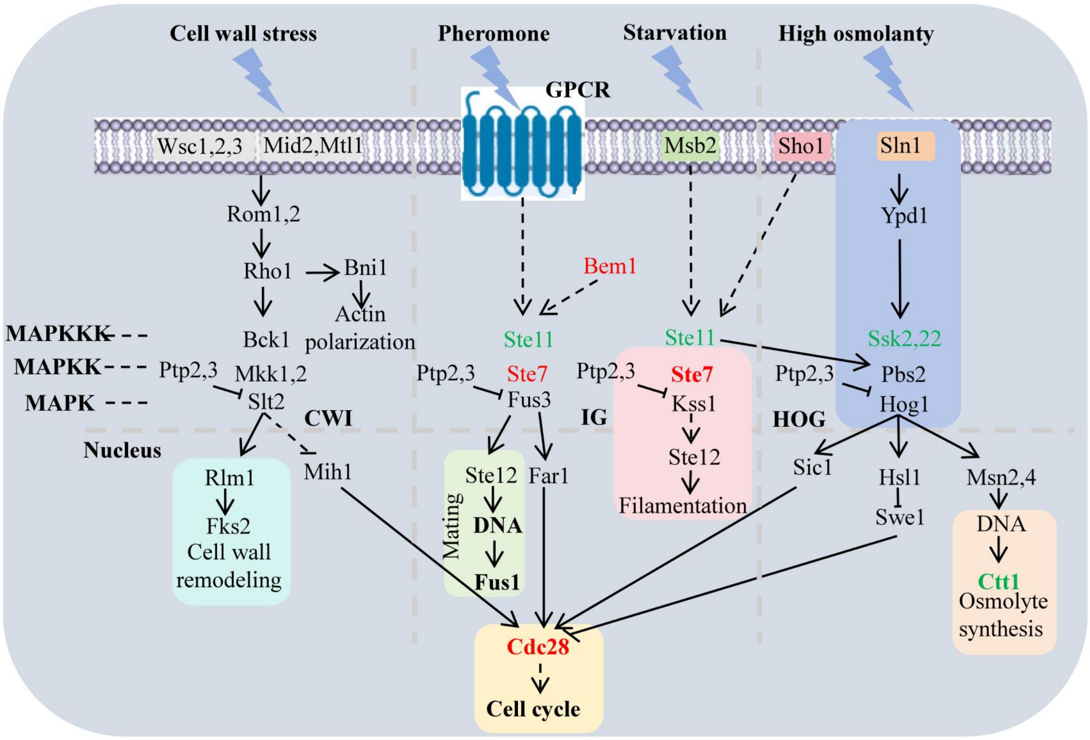

Figure S9 Diagram of the MAPK signaling pathway in B\A group. In the figure, the up-regulation was marked in red, and the down-regulation was marked in green. The solid lines represent direct effects, and the dotted lines represent indirect effects. The arrows represent promotion, and the vertical lines represent inhibition.

Table S1 Table of the media containing different concentrations of chemical epigenetic modifiers used in this experiment. 5-AzaC as the modifier, sterilized water as the solvent, and 0.22 μm filtration membrane were used to filter and remove bacteria.

NO.	Name	Addreviation	Component
1	Potato Dextrose Agar	PDA	200 g potato (peeled and cut into small pieces), 1.0 % glucose, 1.5-2 % agar, deep sea in situ seawater, natural pH, Autoclave (115 ℃, 30 min)
2	Potato Dextrose Agar-50μM 5-AzaC	SDA-50 μM 5-AzaC	200 g potato (peeled and cut into small pieces), 1.0 % glucose, 1.5-2 % agar, deep sea in situ seawater, natural pH, Autoclave (115 ℃, 30 min), 50 μM 5-Aza
3	Potato Dextrose Agar-500 μM 5-AzaC	SDA-500 μM 5-AzaC	200 g potato (peeled and cut into small pieces), 1.0 % glucose, 1.5-2 % agar, deep sea in situ seawater, natural pH, Autoclave (115 ℃, 30 min), 500 μM 5-AzaC
4	Potato Dextrose Agar-1000 μM 5-AzaC	PDA-1000 μM 5-AzaC	200 g potato (peeled and cut into small pieces), 1.0 % glucose, 1.5-2 % agar, deep sea in situ seawater, natural pH, Autoclave (115 ℃, 30 min), 1000 μM 5-AzaC

Table S2 Summary of RNA-seq results.

Abbr.	Sample	Raw reads	Raw bases	Clean reads	Clean bases	Error rate	Q20	Q30	GC pct
A1	0.1 MPa-1	22396653	6.72	21799862	6.54	0.01	98.85	96.83	53.79
A2	0.1 MPa-2	21352469	6.41	20741978	6.22	0.01	98.76	96.71	53.72
A3	0.1 MPa-3	21524383	6.46	20950939	6.29	0.01	98.86	96.91	53.87
B1	40 MPa-1	23669338	7.1	23033329	6.91	0.01	98.86	96.87	54.22
B2	40 MPa-2	26372945	7.91	25827074	7.75	0.01	98.81	96.86	54.27
B3	40 MPa-3	23573959	7.07	22897485	6.87	0.01	98.71	96.61	54.18
C1	0.1 MPa-5-AzaC-1	19644889	5.89	18495678	5.55	0.01	97.48	94.19	52.39
C2	0.1 MPa-5-AzaC-2	17755413	5.33	16534445	4.96	0.01	97.39	94.07	52.1
C3	0.1 MPa-5-AzaC-3	19479882	5.84	18049122	5.41	0.01	97.34	94.09	51.81
D1	40 MPa-5-AzaC-1	23086925	6.93	22253348	6.68	0.01	98.23	95.64	53.29
D2	40 MPa-5-AzaC-2	22030555	6.61	21329774	6.4	0.01	98.62	96.35	53.05
D3	40 MPa-5-AzaC-3	20587774	6.18	19799737	5.94	0.01	98.19	95.49	53.49

Table S3 Table of qRT-PCR primers to validate RNA-Seq results. In gene IDs, up-regulated genes were labeled red and down-regulated genes were labeled green.

NO.	Gene ID	Addreviation	Forward primer	Reverse primer	Length
1	Cluster-4001.939	Ste7	CGACCCCAGACAAAGAGTGG	CACCCAAACGTAGTTTGCCC	236
2	Cluster-4001.2533	Ptp2,3	TTGGATATGCTCAAGCGGCA	AGACATCGTTTGAGTCGCCG	279
3	Cluster-4001.2688	Rlom1,2	CGCTGATCACCTGTCCTGTT	ACAGGGTAATGACCGAGCAA	229
4	Cluster-4001.878	cellulase(CELB)	TCGTGCGAGACTGATGTCTG	AAAAGCAAGGCCTCCACGAA	250
5	Cluster-4001.2338	Sln1	TCGACTCAGGCATGGACTTC	GATGTCACTGTCGCTACGCA	297
6	Cluster-4001.3079	Ste11\Ssk2,22	GGTGTATAGTGGCTCGTCGG	GCGGGTCATCTCTAAGGCTC	190
7	Cluster-4001.893	alpha-amylase (AMY)	GCCACGAGTGGGTATCACAA	GTTTCAGACGTCCACGAGGT	278
8	Cluster-4001.3833	chitinase	CTTTGACGCCACAGTACCCT	GGAGGTGAATGCTGCAATGG	284
